# Cyanobacterial Siderophores—Physiology, Structure, Biosynthesis, and Applications

**DOI:** 10.3390/md17050281

**Published:** 2019-05-10

**Authors:** Erland Årstøl, Martin F. Hohmann-Marriott

**Affiliations:** Department of Biotechnology, PhotoSynLab, Norwegian University of Science and Technology, 7491 Trondheim, Norway; erland.arstol@ntnu.no

**Keywords:** cyanobacteria, siderophore, anachelin, synechobactin, schizokinen, Synechococcus, Anabaena

## Abstract

Siderophores are low-molecular-weight metal chelators that function in microbial iron uptake. As iron limits primary productivity in many environments, siderophores are of great ecological importance. Additionally, their metal binding properties have attracted interest for uses in medicine and bioremediation. Here, we review the current state of knowledge concerning the siderophores produced by cyanobacteria. We give an overview of all cyanobacterial species with known siderophore production, finding siderophores produced in all but the most basal clades, and in a wide variety of environments. We explore what is known about the structure, biosynthesis, and cycling of the cyanobacterial siderophores that have been characterized: Synechobactin, schizokinen and anachelin. We also highlight alternative siderophore functionality and technological potential, finding allelopathic effects on competing phytoplankton and likely roles in limiting heavy-metal toxicity. Methodological improvements in siderophore characterization and detection are briefly described. Since most known cyanobacterial siderophores have not been structurally characterized, the application of mass spectrometry techniques will likely reveal a breadth of variation within these important molecules.

## 1. Introduction

Cyanobacteria rely on iron as an essential cofactor for performing oxygenic photosynthesis and therefore have much larger iron requirements than non-photosynthetic organisms [1]. As iron within cyanobacteria is at a concentration that is 4–6 orders of magnitude higher than in the extracellular environment, the acquisition of iron necessitates a large energetic investment [2]. While iron is one of the most abundant elements on Earth, bioavailable iron in freshwater and marine environments is severely limited. In aqueous environments, bioavailable Fe^2+^ is rapidly oxidized to Fe^3+^ and forms poorly soluble complexes. Fe^3+^ either precipitates as ferric oxyhydroxides or is bound to a wide variety of organic ligands in a “ligand soup” [3]. The concentration of dissolved iron in marine surface waters is variable, falling in the picomolar to a low nanomolar range [4]. In large swaths of the ocean, iron availability is rate-limiting for the growth of cyanobacteria and other phytoplankton. These regions, termed “high-nitrogen, low chlorophyll” (HNLC), have attracted interest as targets for iron fertilization interventions as a way of increasing carbon sequestration and thus potentially counteracting climate change [5,6]. 

Cyanobacteria have developed a number of strategies to survive under iron limitations. Coping mechanisms include remodeling photosynthetic complexes [7], use of iron-accumulating bacterioferritin and replacing iron-containing enzymes with iron-free analogs [8]. Iron import systems are also activated, among them a membrane-bound iron reduction system [9], or an extracellular reduction system that may involve pili [10].

As an alternative import strategy, many cyanobacteria excrete siderophores. Siderophores are biologically produced low-molecular-weight (400–1000 kDA) compounds with an extremely high affinity for iron. Produced by microorganisms to enable iron acquisition under iron-limited conditions, siderophores are widely distributed in bacteria and fungi, and are found in marine, freshwater, and terrestrial environments. Increased iron bioavailability can also be achieved by the complex formation of Fe^3+^ with common metabolites [11] including citric acid [12,13] and humic acids [14]. Typically, siderophores form a strong hexadentate octahedral complex with ferric iron (Fe^3+^). This complex can then be transported inside the cell. The reduction of Fe^3+^ then facilitates its release, as siderophores do not typically chelate ferrous iron (Fe^2+^) nearly as strongly [15]. Based on the primary oxygen-donating ligands that bind the iron, siderophores are divided into four different types [16]. These are the hydroxamates, catecholates, and carboxylates, as well as siderophores with mixed types of these functional groups. All cyanobacterial siderophores that have been identified so far are either hydroxamates or catecholates.

In this mini-review, we will synthesize the current state of knowledge regarding the structure, biosynthesis, uptake mechanisms, detection and biotechnological application of cyanobacterial siderophores. While phytoplankton’s photosynthetic activity is responsible for one third to one half of global carbon sequestration [17], the molecules that phytoplankton produce to cope with growth-limiting iron levels remain somewhat obscure. Most described siderophores stem from terrestrial sources, and it has long been assumed that there is only limited siderophore production by cyanobacteria living in ocean environments. Intuitively, the diffusive loss of the siderophores would make for an energetically costly method of iron sequestration that does not directly benefit the siderophore producer. However, it appears that low-iron, open ocean environments show high concentrations of amphiphilic siderophore variants, that may limit diffusive losses due to association with the cell membrane [18]. Amphiphilic cyanobacterial siderophores are of clear interest in terms of global ocean productivity, but siderophores can also impact more local concerns of microbial cooperation and competition. As we shall see, cyanobacteria use siderophores to outcompete other organisms as antimicrobial agents and to protect themselves from heavy-metal toxicity. These properties make them of biotechnological interest, and concepts have been demonstrated where cyanobacterial siderophores are used for antimicrobial coating materials and for uranium sequestration.

## 2. Hydroxamate Siderophores in Cyanobacteria

**Figure 1:**
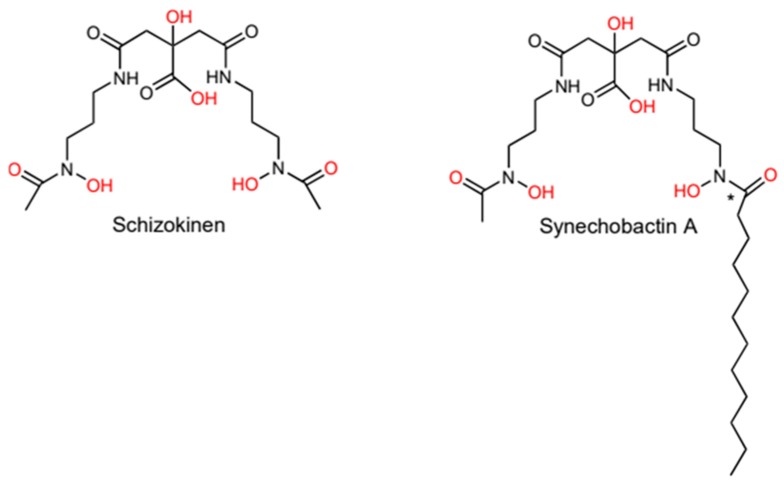
The structures of schizokinen, produced by some *Anabaena* strains, and synechobactin A produced by *Synechococcus* sp. PCC 7002. Iron-binding oxygen-atoms are marked in red. The synechobactins are a suite of siderophores with different fatty-acid chain lengths in the (*) marked position.

Hydroxamate-type siderophores appear to be the most common siderophore type in nature, being widely distributed in both bacteria [19] and fungi [20]. In bacteria, the hydroxamate-moiety is made up of hydroxylated and acylated alkylamines [21]. Many cyanobacteria produce hydroxamates. Most of these siderophores were phenotypically identified in the 1980s and 1990s but have not been explored at a molecular resolution since References [22,23,24,25]. However, two hydroxamate siderophore structures have been determined, schizokinen and synechobactin (Figure 1). These are structurally similar to other dihydroxamate-type siderophores, such as rhizobactin 1021 [26] and aerobactin [27]. Schizokinen is produced by some strains of the freshwater species *Anabaena* sp, strain PCC 6411 and 7120 [24,28]. Schizokinen was first described in *Bacillus megaterium* [29] and is based on citric acid with symmetrical substitutions by amide linkages to a pair of 1-amino-3-(*N*-hydroxy-*N*-acetyl)-aminopropane residues. 

Ferric iron chelation for schizokinen and synechobactin is thought to work by the siderophore forming a hexavalent octahedron with the two oxygens of the two hydroxamate groups, along with the α-hydroxycarboxylate groups of the citrate moiety (marked in red in Figure 1) [24,30]. The stability constants of hydroxamate siderophores vary widely, depending on the number of hydroxamate units, the denticity of the siderophore, and whether the siderophore is cyclic or linear [15]. While no measurements have been made for synechobactin and schizokinen, the structurally similar aerobactin has a stability constant of ~10^23^ [31]. Amphiphilic siderophores do not seem to diverge significantly from otherwise similar non-amphiphilic siderophores in terms of stability constants [32].

### 2.1. Synechobactin is an Amphiphilic Siderophore

The amphiphilic siderophore suite synechobactin has been identified in the marine model organism *Synechococcus* sp. PCC 7002 [33]. Structurally, these siderophores are essentially schizokinen with a fully saturated fatty acid tail on one of the two α-hydroxamate groups (Figure 1). The length of the fatty acid tail varies, with the most common C12, C10 and C8 varieties being named synechobactin A, B, and C, respectively [34]. Additional mass spectrometry studies revealed less common variants with C11, C13, and C14 tail chain lengths [35]. The variability in fatty acid chain length indicates a level of flexibility in the incorporation of fatty-acid tails, with even numbered chain lengths being more common as expected based on the biosynthesis of fatty acids. 

Like many other siderophores based on α-hydroxy-carboxylic acids, the Fe^3+^-synechobactin-complex appears to be photoreactive. Exposing Fe^3+^-synechobactin A to light results in the decarboxylation of the citrate moiety to a 3-ketoglutarate, while the iron remains bound to the siderophore [34]. Siderophores from other bacteria also mediate photoreduction of Fe^3+^ to Fe^2+^ [36,37], making this a potential mechanism for Fe^3+^-reduction in *Synechococcus* sp. PCC 7002. However, it is unlikely that this light-driven reduction is the same reduction mechanism that underlies the previously reported reduction of Fe^3+^ in *Synechocystis* PCC 6803, as this organism is not known to produce siderophores [38]. It is not clear how the photoreactivity of synechobactin impacts the import of siderophores into the cyanobacterial cell. In the case of the similar aerobactin, the photoproduct-Fe^3+^-complex appears to have similar properties as the original siderophore, and both variants can be utilized by the host bacteria for iron acquisition [39]. While schizokinen contains the same α-hydroxy-carboxylic acid as synechobactin, no photoreactivity has been shown for this siderophore. 

The fatty acid tail of amphiphilic siderophores is thought to modulate association with the cell membrane, with the strength of association increasing with the length of the fatty acid tail and decreasing with the degree of saturation. Most other marine amphiphilic siderophores that have been isolated are similar to each other, being peptide-based hydroxamates. These include marinobactin from *Marinobacter* sp, aquachelin from *Halomonas aquamarina* [40] and amphibactin from *Vibrio* sp. R-10 [41]. Amphiphilic siderophores have also been found in oil-associating bacteria after oil spills [42,43], implying some role in role in iron acquisition in this environment. Marinobactins and aquachelins can form micelles even in low concentrations, which shrink in diameter when they coordinate with Fe^3+^, and can furthermore form vesicles when exposed to excess Fe^3+^ [40]. The pathogenic bacterium *Acinetobacter haemolyticus* produces acinetoferrin, a citrate-based dihydroxamate similar to synechobactin, but with two *trans*-2-octenoyl hydrocarbon chains. When bound to iron, acinetoferrin shows a 30-fold loss of membrane-affinity and an increased tendency to flip-flop across lipid bilayers [44]. 

The length of the saturated synechobactin fatty acid tails ranges from C8 to C14. This is shorter than the C14 to C18 fatty acid moieties of the cell-associated amphibactin and equivalent to the C8 to C14 fatty acid moieties of marinobactin. Marinobactins are predominantly found in the growth medium under isolation procedures [41,45]. Similarly to marinobactin, the suite of different fatty acid chain lengths in synechobactin likely indicate some gradient of cell-association. Synechobactins have been found in both media and the cell pellet isolates of cultured *Synechococcus* sp. PCC 7002 [34]. How synechobactins act in terms of micelle formation and membrane affinity in iron-bound and iron-free forms has not been explored.

So far, synechobactin is the only cyanobacterial amphiphilic hydroxamate siderophore that is characterized to a structural level. Synechobactins or other membrane-anchored siderophores may, however, also be present in other cyanobacteria. For example, *Anabaena cylindrica* strains 7122 and 1611 produce hydroxamate siderophores that remain in the organic phase after solvent extraction and have reduced polarity compared to schizokinen [24].

### 2.2. Schizokinen and Synechobactins Are Synthesized by NIS-Based Systems

The biosynthesis of siderophores involves either a combination of non-ribosomal peptide synthases (NRPS) and polyketide synthase (PKS) or NRPS independent synthases (NIS). Citrate-based siderophores such as schizokinen and synechobactin are produced by the latter system, which will be briefly described here. 

While the NIS pathways function primarily in the synthesis of polycarboxylate siderophores, the synthesis of hydroxamates and mixed-type siderophores by NIS has also been shown, with the genetic components predominantly found in actinobacteria and proteobacteria [46]. Typically, NIS biosynthesis pathways of siderophores contain at least one enzyme with conserved N-terminal iron uptake chelate (Iuc A/Iuc C) domains, with a C-terminal domain used for iron metabolism or transport. NIS synthases can be categorized by the formation of a peptide bond between a hydroxamate-amine with a carboxylic acid substrate—Citric acid (type A and A’ NIS), α-ketoglutaric acid (type B NIS)—or a succinic- and citric acid derivative (type C and C’ NIS) [46,47]. NIS-enzymes are responsible for a single enzymatic reaction, typically the activation of citric acid by adenylation, and nucleophilic capture of alcohol or amine groups, which with the release of AMP produces a citryl intermediate. Many siderophores require more than one NIS-enzyme for biosynthesis [46]. NIS-synthases catalyzing the peptide bond formation with citric acid (Iuc A), ana-ketoglutaric (Acs A) acid and succinic acid (Iuc C) have been investigated in detail. It has been proposed that elongation and circulation of siderophores are accomplished by two NIS domains that interact with the two substrates, with each substrate potentially being a product of previous NIS-synthase reactions [47].

The aerobactin biosynthesis of *E. coli* K-12 is considered prototypical, and bioinformatic analysis for NIS-produced siderophores is often based on a similarity to the i*ucA* gene that was characterized in the aerobactin pathway [46]. In aerobactin synthesis, IucA catalyzes the condensation of *N*-acetyl-*N*-hydroxylysine and citric acid to form *N*-citryl-acetyl-*N*-hydroxylysine. IucC mediates the addition of another *N*-acetyl-*N*-hydroxylysine to the citric acid moiety of *N*-citryl-acetyl-*N*-hydroxylysine to form the symmetric aerobactin molecule [27].

There is currently no biochemically-substantiated biosynthesis pathway established for the cyanobacterial siderophore schizokinen. However, schizokinen is an intermediary product in the biosynthesis of another siderophore, rhizobactin 1021. (Rhizobactin 1021 is unrelated to the siderophore rhizobactin). The synthesis of rhizobactin 1021 is similar to the synthesis of aerobactin, as a hydroxylated and acetylated molecule is first combined with citric acid and the resulting product is combined with another hydroxylated and acetylated molecule to form a symmetrical siderophore. Rhizobactin 1021 is synthesized from l-glutamic acid and l-aspartic b-semialdehyde which combines to form 1,3-diaminopropane. Further hydroxylation and acetylation lead to *N*4-hydroxy-1-aminopropane, and two of these molecules are bound to citrate to form schizokinen [48]. Since schizokinen is an intermediary in the rhizobactin 1021 synthesis, it is likely that schizokinen synthesis shares an enzymatic pathway with the rhizobactin 1021 synthesis. This idea is substantiated by the identification of a cluster of genes (*all0390*, *all0392*, *all0393*, and *all0394*) in *Anabaena* sp. PCC 7120 with a similarity to the biosynthesis-genes for rhizobactin 1021 [49]. Transcription of *all0390* is induced by iron limitation [50].

While no study has looked specifically into the biosynthesis of synechobactins in *Synechococcus* sp. PCC 7002, IucA/IucB/IucC-family proteins have been identified in an 8-gene operon (G0025–G0018, named *sidA* to *sidH*) [51]. The operon in question is upregulated under iron limitation conditions [52,53]. The biosynthesis of synechobactin is likely an extension of the schizokinen synthesis outlined above. Rhizobactin 1021 incorporates a trans-unsaturated fatty acid as a replacement of one acetic acid in schizokinen. Similarly, the synechobactin suite of siderophores is likely produced by the incorporation of a variety of saturated fatty acids in this final step. Chromatographic ^56^Fe peaks that correspond with schizokinen have been found in growth-phase *Synechococcus* sp. PCC 7002, supporting this idea [35].

## 3. (Hydroxamate) Siderophores Can Bind Other Metals

Siderophores have the capability of complexing with metals other than iron. This feature can be used by microorganisms either for the uptake of essential metals like manganese or zinc, or it can sequester toxic metals outside of the cell. Coordination-chemistry of siderophores with non-iron metals is in most cases underexplored as it is typically not investigated when new siderophores are discovered. In general, the metal binding of siderophores is governed by the hard-soft acid-base theory. In these terms, the hard oxygen atom donors of siderophores are not necessarily well suited to bind to softer metals like Cu(II) or Zn(II), leading to lower affinities [54]. Nevertheless, cyanobacterial siderophores have been shown to interact with other metals in biologically and potentially technologically meaningful ways.

In cyanobacteria, hydroxamate siderophores have been shown to bind to copper, and siderophore cycling may have a role in preventing copper toxicity [23,55]. Under conditions of high copper but low iron, schizokinen functions as a copper chelator in *Anabaena* sp PCC 7120, reducing the toxicity of the metal, while a siderophore-independent system appears to be largely responsible for iron uptake. Schizokinen thus appears to have a double functionality in this organism [56]. In another case, the chelation of copper appears to play a role in microbial competition: the hydroxamate siderophores from *Anabaena flos-aquae* have been shown to inhibit the growth of the alga *Chlamydomonas reinhardtii* [57]. The effect is independent of the iron level available to the algae, instead of appearing to rely on siderophoric chelation of cobalt and copper.

Complexation of siderophores with metals other than iron has raised interest in the use of siderophores for heavy-metal sequestration. This can be done by combining siderophore-producing bacteria with the growth of metal-resistant plants [58]. Hydroxamate siderophores from *Synechococcus elongatus* BDU 130911 have been shown to complex with uranium [59]. Interestingly, as long as uranium is present, siderophore production is maintained in this organism even under iron-replete conditions, indicating some biological role of the siderophore in the detoxification of heavy metals. Similarly, the rice paddy-field cyanobacterium *Anabaena oryzae* produces a hydroxamate-type siderophore that can chelate Cd^2+^ even under iron-replete conditions [60].

## 4. Catecholate Siderophores

Catecholates are siderophores characterized by one or more iron-binding catechol moieties: the *ortho*-isomer of dihydroxybenzene.

The catecholate siderophore that has been studied in most detail is enterobactin, first isolated from *Salmonella typhimurium* and *Escherichia coli* [61,62]. Consisting of three catecholate-moieties bound by amide-linkages to a trilactone backbone, enterobactin binds iron by forming a hexadentate tri-catecholate [63]. Catechol binding chemistry with iron is generally flexible, allowing mono, bis and tris catechol-Fe^3+^ complexes, depending primarily on pH and iron concentration [64,65]. The tris catechol-Fe³^+^ complexes have remarkably high iron affinities, with enterobactin-Fe^3+^ having a stability constant of 10^49^ [66]. Catechol functional groups are generally stronger iron chelators than hydroxamate-groups, but are more pH-sensitive. This is due to the number of protons displaced by iron binding: iron binding displaces two hydrogens for a catechol functional group, but only one for hydroxamates [15].

**Figure 2:**
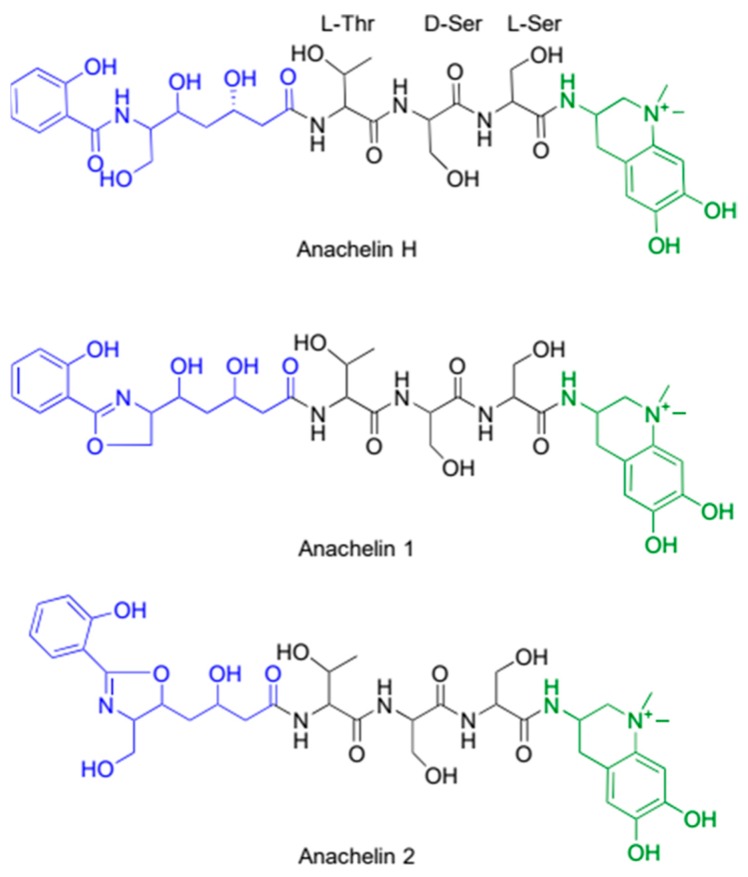
The structures of the catecholate siderophores anachelin, which are produced by *Anabaena cylindrica*. Anachelins have a tripartite structure, composed of a polyketide (blue), a tripeptide (black) and an alkaloid containing the iron binding catechol moiety (green).

In cyanobacteria, catecholate-type siderophores have been discovered in both marine *Synechococcus* strains, and in freshwater *Anabaena*-strains [25,36]. The only cyanobacterial catecholate siderophore that has been structurally characterized is anachelin from *Anabaena cylindrica* [67]. Anachelins have a somewhat unusual structure, featuring a tetrahydroquinolinium-derived chromophore with a catechol diamine. This unique alkaloid is bound to a polyketide through a tripeptide consisting of l-Thr, d-Ser, and l-Ser. Anachelin occurs in different forms (Figure 2), where the most common form is named anachelin H, and structural isomers of a dehydration reaction termed anachelin 1 [67] and anachelin 2 [68]. Two ester forms have also been identified. Anachelin H contains a terminal salicylamide in the polyketide-part, while anachelin 1 and 2 are terminated by an oxazoline ring. It is likely that anachelin H is the biologically relevant product, while anachelin 1 and 2 are formed under dehydrating conditions [69]. At physiological pH, mono, bis, and tris anachelin-iron complexes can occur [70], but which of these is prevalent under low iron concentrations is unknown.

### 4.1. Anachelin Is Produced by Non-Ribosomal Peptide Synthase Pathway for Siderophores

**Figure 3:**
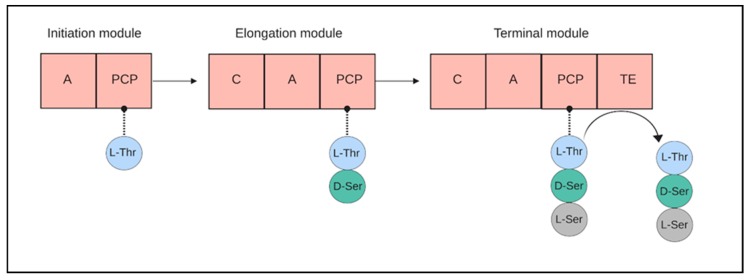
A cartoon demonstrating synthesis of peptide chains by non-ribosomal peptide synthases (NRPS) by a sequence of modules. Amino-acids are activated by the adenylation domain (A), transferred by the peptidyl carrier domain (PCP) and bound to each other by condensation domain (C). A final thioesterase (TE) domain releases the peptide. (Created with Biorender.com).

Peptide-based siderophores such as anachelin are often produced by a combination of the systems combining NRPS and polyketide synthases (PKS). NRPS pathways are well studied as they form the biosynthetic basis of a wide variety of peptidic secondary metabolites [71,72,73,74]. The NRPS pathways are typically composed of multi-domain peptide synthases, where a peptide bond formation between the amino acids is catalyzed by consequent modules [75]. Each amino acid bound to the chain requires a separate module, which is composed of three domains: The amino acids are activated by the adenylation domain (A), while monomers are transferred by the peptidyl carrier domain (PCP). A condensation (C) domain then forms the peptide bonds between the amino acids. In the end, the mature oligopeptide is released by a thioesterase (Te) domain (Figure 3). Often, NRPS is coupled with the activity of polyketide synthases (PKS) to create more complex structures [76].

Structural and functional similarities connect the evolution of polyketide synthases to that of NRPS and even closer to fatty acid synthases. However, while one fatty acid synthases repetitively add the two carbon skeleton from the C3 precursor malonyl-CoA under release of CO_2_, several PKS modules—each with a potentially additional enzymatic capability—can be combined within a single protein, similar to NRPSs. These modules can also have an extended range of substrates, including propionyl-CoA and methylmalonyl-CoA [77]. The other major differences between fatty acid synthesis and PKS synthesis is that in fatty acid synthesis the keto-group is reduced to obtain a saturated hydrocarbon backbone, while in the PKS-mediated condensation product, the keto group remains, but may be modified if the respective PKS module possesses additional enzymatic functions. These keto- or derived hydroxy-groups can serve as potential ligands for Fe^3+^.

The three-part structure of anachelin is the result of a combination of NRPS and PKS synthesis pathways. A biosynthetic pathway for the peptide and polyketide part of anachelin inspired by the NRPS/PKS assembly of yersiniabactin produced by *Yersinia pestis* has been proposed [78,79]. It is suggested that starting on the polyketide part of the molecule, l-Ser condenses to salicylic acid on an initial NRPS before cyclization, forming an oxazoline ring. Switching to a PKS-module, this intermediate is elongated by the condensation of two malonyl groups. After ketone reduction, a second NRPS attaches the peptide sequence l-Thr-d-Ser-l-Ser.

The final synthesis of the catecholate chromophore part of anachelin has attracted some interest due to the unique tetrahydroquinolinium chromophore. A C-terminal tyrosine is added to the precursor molecule and is subsequently converted to a dopamine derivative. Enzymatic oxidation by a tyrosinase into an *ortho*-quinone before aza annulation results in the final anachelin chromophore. Full chemical synthesis of anachelin has been achieved by a biomimetic route [80].

A bioinformatic analysis has identified a cluster of 20 genes in *Anabaena cylindrica* PCC 7122 that is likely related to the synthesis of anachelin. In addition to NRPSs that correspond to the l-Thr-d-Ser-l-Ser structure, four candidate genes for the creation of the salicylate starter unit, 2 PKSs that could add malonyl groups, as well as a putative hydrolase coupled with other genes necessary for the creation of the catechol moiety have been assigned. The gene cluster additionally codes for a thioredoxin-like protein, a siderophore-binding protein and a siderophore receptor [73]. Taken together, the gene cluster is likely to accommodate the hypothesized biosynthesis pathway for anachelin.

### 4.2. Antimicrobial Properties of Anachelin

The tripartite structure of anachelin invites the question of the functionality of the polyketide fragment, as it does not seem to be directly involved in iron chelation. Anachelin exhibits an inhibitory effect on the growth of the competing green algae *Kirchneriella contorta* and *Chlamydomonas reinhardtii* [81]. This effect is found even under iron-replete conditions, rendering an “iron monopoly” explanation unlikely. The allelopathic effect of anachelin was found to be dependent on the polyketide fragment. However, evaluation of anachelin against a variety of microbes found a limited antibacterial activity [82].

Siderophores interface with metal surfaces, which gives microorganisms an anchor to colonize these surfaces. The siderophore pyoverdine plays an important role in biofilm-initiation by *Pseudomonas aeruginosa*, demonstrating the first case of covalent bonding between a substrate and the cell surface [83]. Siderophore adsorption to metal oxide surfaces has been shown by the *E. coli*-produced enterobactin [84] substantiating that biofilm formation is a possible function for siderophores. This interaction between siderophores and metal surfaces has been demonstrated in preventing biofilm formation. The iron-oxide binding properties of anachelin and its derivatives have attracted interest for their use as anchor-molecules for an antifouling agent. The strong binding of catechol siderophores to metal oxide surfaces represents a suitable anchor to connect other molecules such as the antifouling agent PEG. This has been demonstrated by synthesizing a PEG-anachelin-complex and applying it to a TiO_2_ surface with a simple dip-and-rinse procedure [85,86]. Similarly, the anachelin-PEG complex has also been fused with an antibiotic. Here the anachelin-PEG is used as a bridge between a titanium-oxide surface and the antibiotic vancomycin. The tested coatings have good stability over multiple wash cycles and show continued anti-biological activity [87].

## 5. Siderophore Cycling

**Figure 4:**
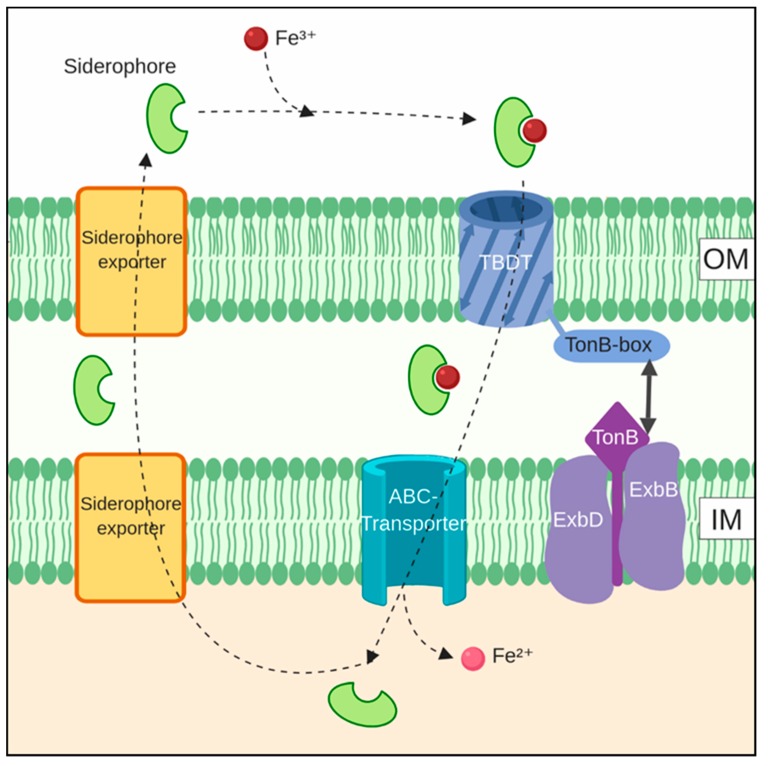
An overview of siderophore cycling in a generic organism. Typically, iron-loaded siderophores are imported through TonB-dependent transporters (TBDT) in the outer membrane, with power supplied by a TonB-ExbB-ExbD-complex in the inner membrane. Further transport is done by ABC-transporters. Export of siderophores can happen by a variety of transporter types. Note that the iron is reduced to Fe^2+^ at some point during this cycle, but the timing and location of this is often not clear. Created with Biorender.com.

Siderophore cycling has two components: (1) the export of siderophores and (2) the import of the iron-loaded siderophore [88]. Presumably, siderophores are recycled and used multiple times by the producing organism or community. The extent of recycling is an intractable problem experimentally, and it is thus often only stated that siderophores are recyclable, without any investigation of the actual cycling. In the case of photoreactive siderophores such as synechobactin, the chemical change induced by light permanently changes the siderophore, making its recycling difficult to envisage. In the case of more complex siderophores such as anachelin, the energetic investment per molecule makes recycling more likely. A scheme showing the overall cycling of siderophores in a typical organism is shown in Figure 4.

### 5.1. Siderophore Export

Knowledge of siderophore export, especially in cyanobacteria, is limited. Three different protein types are involved in the siderophore export process in various bacteria: the major facilitator superfamily (MFS), resistance nodulation and cell division (RND) superfamily, and the ATP-binding cassette (ABC) superfamily [89].

The only characterized siderophore export system in cyanobacteria is that of *Anabaena* sp. PCC 7120. In this organism, the inner membrane MFS protein SchE (*all4025*) is required for the secretion of schizokinen, along with the TolC-like outer-membrane protein hgdD (*alr2887*), with the two proteins likely working in tandem [56].

### 5.2. Siderophore Import

The import of iron-loaded siderophores is broadly similar across many bacteria and is illustrated in Figure 4. The transport of incoming siderophore-iron complexes into the periplasmic space is enabled by receptor proteins, TonB-dependent transporters (TBDT) located in the outer membrane. TBDTs consist of a β-barrel domain and a “plug” domain in the barrel interior that acts in concert with a TonB-box on the periplasmic side. Energy for transport is derived from the proton-motive force and is mediated through the association of TonB with inner membrane proteins ExbB and ExbD, forming a TonB-ExbB-ExbD-complex. Once in the periplasm, Fe^3+^-loaded siderophores are transported across the inner membrane, often by ABC-transporters [90].

Once inside the cell, iron has to be removed from the siderophore. This can happen either through the reduction of iron from Fe^3+^ to Fe^2+^ by ferric siderophore reductases or by ferric-siderophore hydrolases [89] or by the photolysis of iron-bound siderophores, as described for synechobactin. In the case of photolysis, whether the reduction event takes place outside or inside the cell is not clear.

In *Anabaena* sp. PCC 7120, the import of iron complexed schizokinen across the outer membrane is accomplished by two TBDTs, named the schizokinen transporter (*alr3097*, SchT) [49] and IutA2 (*alr2581*). Transcription data indicate that IutA2 is transcribed earlier in the iron starvation response than SchT [50].

The TBDTs are likely dependent on the inner membrane complex TonB3-ExbB3-ExbD3 (*all2585-all5047-all4056*) for energy. The genes are upregulated under an iron limitation, while similar genes show distinct regulation pattern under other conditions. Additionally, deletion mutations of the genes show an iron starvation phenotype [88].

In the periplasm, Fe-schizokinen is recognized and transported to the cytoplasm via the hydroxamate system FhuBCD (*all0387*, *all0388*, and *all0389*, respectively). These genes are similar to FhuBCD in *E. coli*, where FhuD functions as a periplasmic binding protein, FhuB is a membrane-embedded transporter and FhuC is an ATP-binding protein. Interestingly, while all three genes are upregulated under an iron limitation, they are regulated independently, as *fhuC* responds differently than *fhuB/D* to changing copper and citric acid levels. The significance of this is not clear [50,88].

The release mechanism for ferric iron bound to schizokinen has been found to involve a ferric siderophore reductase in other bacteria [91].

TBDTs are unevenly distributed in cyanobacteria. In general, TBDTs seem to outnumber the amount of TonB-proteins, indicating some functional flexibility in TonB-complexes. The genome of *Anabaena* sp PCC 7120 contains 22 TBDTs, while no candidate genes were identified in the marine *Prochlorococcus. Synechococcus* sp PCC 7002 contains 6 TBDTs, with two found by sequence similarity to resemble known schizokinen transporters, and two found to resemble hydroxamate transporters more broadly [92]. As TBDTs function in the uptake of a variety of compounds [90], variability in TBDTs cannot be explained purely in terms of siderophore or iron uptake more broadly.

The large number of TBDTs in some organisms is partially explained by the possibility of uptake of siderophores which are not produced by the organism (xenosiderophores), known as siderophore piracy. *Anabaena* sp PCC 7120 has been shown to make use of siderophores such as aerobactin [24] and the tris-hydroxamate desferroxamine B (DFB). Aerobactin is likely taken up by the same TBDTs as schizokinen, but DFB is more dissimilar to schizokinen and is transported across the outer membrane by an unidentified TBDT. The import of different siderophores appears to converge on the use of FhuBCD across the inner membrane [50,93].

Similarly, the non-siderophore-producing *Synechocystis* sp. PCC 6803 is capable of utilizing both schizokinen and the unnamed siderophore of *Anabaena variabilis* ATCC 29413 and can grow with iron-siderophore complexes as their only source of iron. While reductive iron uptake is considered the primary means of iron acquisition for this organism, the genome of *Synechocystis* has been found to code for a singular *tonB (slr1484*) gene, which functions along with exbB1–exbD1 (*sll1404, sll1404*) in apparently typical siderophore uptake [94]. Additionally, siderophore uptake in *Synechocystis* sp. PCC 6803 is dependent on TBDT SchT (*sll1206*), which is organized in a gene cluster along with the gene coding for the ABC-type transporter FecB1CDE (*slr1316* to *slr1319*). This system is able to operate independently of the ferric and ferrous iron transporters that are presumed to be the main iron acquisition mechanisms in *Synechocystis sp. PCC 6803* [95].

## 6. The Distribution of Siderophores in Cyanobacteria

**Figure 5:**
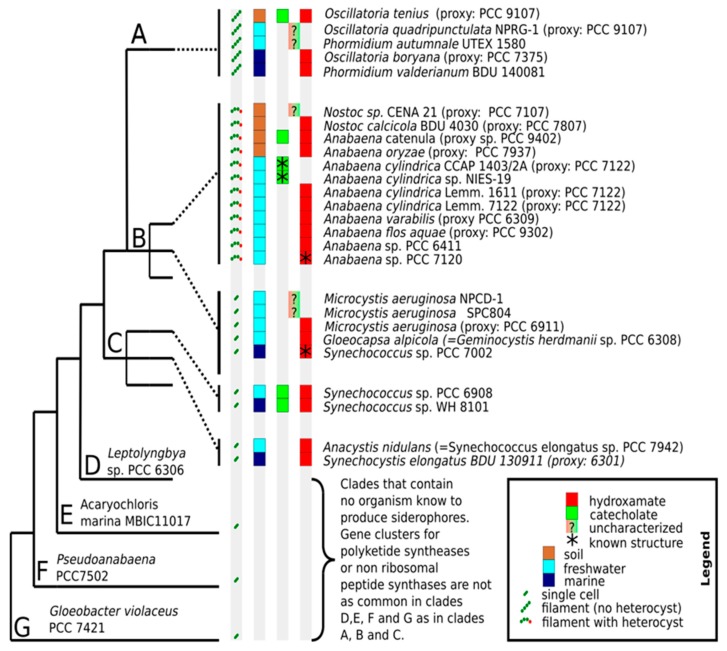
The overview of known siderophores with associated organisms, cell morphology, habitat and phylogenetic association. The type of siderophore is only determined structurally in a few organisms (indicated by asterisks). The phylogenetic class designations (**A**–**G**) from References [96] have been used. In classes where no siderophore-producing organisms are known, representative species are indicated. A question mark (“?”) indicates that the type of siderophore is not known. Species names of the original studies are shown, with updated species names indicated by “=”. Where genetic data is not available, proxy-species with known phylogenetic positions were assigned based on described characteristics of the organism.

The number and structural diversity of siderophores produced by cyanobacteria are probably much higher than the low number that structurally characterized siderophores may imply. The phylogenetic and environmental distribution of known siderophore-producing cyanobacteria is shown in Figure 5. Phylogenetically, siderophores are widely distributed in cyanobacteria, but so far have not been identified in the earliest-branching cyanobacterial clades, which also contain fewer NRPS and PKS coding genes than later branching clades [96]. In terms of the environment, Figure 5 shows that siderophores are produced by unicellular, filamentous and heterocyst-forming cyanobacteria in terrestrial, freshwater and marine environments. The apparent dominance of fresh-water organisms in the figure may not represent an unbiased assessment. Testing of four different marine cyanobacteria, unicellular *Synechococcus elongatus* BDU 130911, filamentous heterocystous *Nostoc calcicola* BDU 40302, as well as the filamentous non-heterocystous cyanobacteria *Phormidium valderianum* BDU 140081 and *Oscillatoria boryana* BDU 141071 established that under iron limitation, all four of them produce siderophores [59]. This study highlights the presence of siderophores in a wide variety of marine cyanobacteria, and that the sparseness of reports on marine siderophore-producing cyanobacteria may be due to sampling bias.

In terms of distribution between the two biosynthetic pathways for siderophore production, siderophores based on NIS do not seem to be widely distributed in cyanobacteria. Only in *Anabaena* sp. PCC 7120, *Anabaena variabilis* ATCC 29413 and *Synechococcus* sp. PCC 7002 have NIS been tentatively identified in available genomes, while NRPSs were identified in more than 50% of analyzed cyanobacterial genomes [97]. However, the aforementioned study fails to find either putative NIS or NRPS coding genes in known siderophore-producing organisms such as *Synechococcus elongatus* PCC 7942, raising the possibility of false negatives. At any rate, it appears that the cases of schizokinen and synechobactins are not particularly representative for the biosynthesis of siderophores in cyanobacteria. While NRPSs and PKSs are prevalent in cyanobacteria, they are classes of enzymes that participate in the synthesis of many non-siderophore molecules [72,74]. It is thus difficult to unambiguously identify siderophore production based on genomic analysis. However, some gene clusters have been identified in addition to the anachelin cluster noted above. Genetic deletion indicates that a gene cluster that encodes 24 open reading frames, including open reading frames for seven NRPSs and two polyketide synthases, are responsible for the majority of siderophore production in *Anabaena* sp. strain PCC 7120 [98]. This siderophore is different from the schizokinen produced by the same organism. An ortholog to this cluster is found in *Nodularia* sp. CCY 9414 [73]. The structure of the siderophore(s) produced by these gene clusters is not known. Analysis of 50 Brazilian freshwater cyanobacterial strains showed siderophore production in four isolates, all of which contained NRPSs and PKSs, but no test was made for NIS-based synthesis [99].

References indicating the presence of siderophores in Figure 5 are (organisms in alphabetical order): *Anabaena catenula* (UTEX 375) [25], *Anabaena cylindrica* NIES-19 [56], *Anabaena cylindrica* CCAP 1403/2A [55], *Anabaena cylindrica* Lemm. 1611—[22,24], *Anabaena cylindrica* Lemm. 7122 [33], *Anabaena flos-aqua* [22,100], *Anabaena oryzae* [60], *Anabaena* sp. PCC 6411 [24], *Anabaena* sp. PCC 7120 [24,28], *Anabaena variabilis* [68,101,102], *Anacystis nidulans* [22], *Gloeocoapsa alpicola* [22], Microcystis aeruginosa [22], *Microcystis aeruginosa* NPCD-1 [99], *Microcystis* sp. SPC804 [99], *Nostoc calcicola* BDU 4030 [59], *Nostoc* sp. CENA21 [99], *Oscillatoria tenius* [25,103], *Oscillatoria boryana* BDU 140791 [59], *Oscillatoria quadripunctulata* NPRG-1 [99], *Phormidium autumnale* UTEX1580 [99], *Phormidium valderianum* BDU 140081 [59], *Synechococcus elongatus* BDU 130911 [59], *Synechococcus* sp. PCC 6031 [34], *Synechococcus* sp. PCC 6908 [34], *Synechococcus* sp. PCC 7002 [40,41,42], *Synechococcus* sp. PCC 7942 [34,104], *Synechococcus* sp. WH 8101 [34].

## 7. Identification Methods

Methodology development has allowed considerable improvements in siderophore discovery and characterization in recent years, as well as improvements in analyzing metal-ligand interactions in ocean water. Traditionally, the detection of siderophores has been based on chemical properties of the active group by assays such as the Csaky assay [105] and the Atkin assay for hydroxamates [106,107] and the Arnow test for catecholates [108]. The widely used chromeazurol S assay (CAS-assay) allows semi-quantitative siderophore identification by a color change in the presence of stronger iron chelators than CAS itself [109] and can be performed either in liquid media or on agar plates. Extending the assay methodology allows high-throughput siderophore detection by microplates [110] and detection independent of the species’ ability to grow on CAS-agar [104]. However, the detection limit of the CAS-assay is higher (~80 nmol/L) than for modern voltammetric methods (~3 nmol/L) [111]. The sensitivity limitation of the CAS-assay makes it likely that testing for cyanobacterial siderophore production, especially without a properly induced iron limitation, has resulted in false negatives.

For elucidating structural information of siderophores, nuclear magnetic resonance spectroscopy (NMR), X-ray crystallography and mass spectrometry methods have all been employed. Both schizokinen and synechobactin were originally characterized primarily by NMR spectroscopy [29,34], but this method requires a large sample and long analysis times. With better knowledge of siderophore structural elements and technological advances, most analyses of siderophores are performed by mass spectrometry that requires small samples and can resolve iron-complexes. Structural characterization can be performed by mass spectrometry approaches such as electrospray ionization mass spectrometry (ESI-MS) [112,113,114].

Direct detection of siderophores from seawater samples is hindered by low concentrations of both iron and iron chelators. One approach for the rapid screening of seawater samples makes use of HPLC–tandem inductively coupled plasma-mass spectrometry (HPLC–ICP-MS). Requiring minimal sample preparation, the method can quickly detect metal-ligand complexes [115]. Significant sensitivity increases for this method have been achieved by the introduction of a hexapole collision cell which minimizes ^40^Ar^16^O^+^ interference with ^56^Fe and with the addition of oxygen to the sample carrier gas, which ensures the complete combustion of organic solvents. These and other measures lower the detection limits to 30–250 femtomoles for iron complexes [116].

Combining different MS-methods allows sensitive detection, quantification, and characterization of organic-metal complexes. Using both inductively coupled plasma mass spectrometry (ICP-MS) and high-resolution electrospray ionization mass spectrometry (HR-ESI-MS) has been demonstrated in the detection and characterization of synechobactin from *Synechococcus* sp. PCC 7002 [35]. Running liquid chromatography in tandem with ICP-MS, organic-iron complexes elute from the column, and the iron is directly ionized and detected, giving a retention time and abundance of each iron-complex. Use of the soft ionization technique HPLC-ESI-MS then gives masses of intact metal complexes and can be coupled with MS^2^ fragmentation patterns to structurally characterize the compounds. This combined methodology revealed the presence of synechobactin-variants with odd-numbered fatty acid tails, which previously went undetected by NMR-based methods.

Increased speed and precision in determining siderophores has been achieved by a newly developed 21 T Fourier Transform Ion Cyclotron Resonance Mass Spectrometer (FTICR MS) in combination with electrospray ionization [117]. In this approach, identification is based on the high accuracy of molecular mass assignments and isotopic patterns of both iron and other elements.

Imaging mass spectrometry (IMS) by matrix-assisted laser desorption ionization-time of flight (MALDI-TOF) has been used to evaluate the spatial distribution of cyanobacterial siderophores colonies grown on agar [118]. This technique may open a window for exploring siderophore cycling within complex biofilms in the future.

## 8. Conclusions

Considering the ecological significance of iron uptake by plankton, it is surprising that so few cyanobacterial siderophores have been characterized. As cyanobacteria are expected to be responsible for ~25% of marine primary production [119], identifying the breadth of chemistry employed to deal with growth limiting iron deprivation should be prioritized. As iron complexed to organic ligands is the primary form of iron in the ocean, characterizing siderophores and their chemistry is crucial to our understanding of the marine iron cycle. This is especially important to make reasoned predictions concerning the effect of ocean acidification on iron availability and, consequently, carbon sequestration.

Our review highlights the emerging understanding of cyanobacterial siderophores in the ocean environment, as freshwater species were mostly used to understand siderophore biosynthesis and siderophore cycling. The discovery of the synechobactins from *Synechococcus* sp. PCC 7002, which can likely be anchored to a membrane, voids the argument that siderophores impose an unrecoverable cost to cyanobacteria in ocean environments. Additionally, genetic analyses show that the IucA/IucC homologs used to produce synechobactin and schizokinen are not widely distributed in cyanobacteria. This makes it likely that the wide variety of marine cyanobacteria that produces hydroxamate-type siderophores do so in ways that are not well-represented by the research that has been done so far.

The development and application of mass spectrometry techniques have significantly increased the ease with which siderophores can be identified and characterized to gain further insight into the distribution, physiology and structural characteristics. In recent years, cyanobacterial siderophores have been identified in studies with a focus on “proof-of-technological-concept”, rather than an interest in the biosynthesis and physiological role of the molecule as a target itself. Application of these technologies to non-model organism cyanobacteria will surely lead to novel compounds with ecological as well as technological significance. The unique structure of anachelin and the nascent applications based on this molecule demonstrate that the potential of cyanobacterial siderophores matches their ecological importance.
